# Mind the Gap! Sleep Problems in Children With ADHD—A Qualitative Analysis of Clinician Training Needs

**DOI:** 10.1111/cch.70254

**Published:** 2026-03-11

**Authors:** Lucy Smith, David Daley, Samuele Cortese, Catherine M. Hill, the DISCA team

**Affiliations:** ^1^ NTU Psychology, School of Social Sciences Nottingham Trent University Nottingham UK; ^2^ Centre for Innovation in Mental Health (CIMH), School of Psychology, Faculty of Environmental and Life Sciences University of Southampton Southampton UK; ^3^ School of Clinical and Experimental Sciences (Paediatric Neuroscience), Faculty of Medicine University of Southampton Southampton UK; ^4^ Hampshire and Isle of Wight Healthcare NHS Foundation Trust Southampton UK; ^5^ New York University Child Study Center Hassenfeld Children's Hospital at NYU Langone New York City New York USA; ^6^ DiMePRe‐J—Department of Precision and Rigenerative Medicine‐Jonic Area University of Bari ‘Aldo Moro’ Bari Italy; ^7^ Paediatric Sleep Medicine Southampton Children's Hospital Southampton UK

**Keywords:** attention deficit hyperactivity disorder, clinicians, developing digital interventions, sleep difficulties

## Abstract

**Background:**

This study aims to explore for the first time the knowledge, understanding and management of sleep problems in children with ADHD among clinicians who specialise in sleep and ADHD. The aim was to inform the development of digital sleep awareness training for clinicians.

**Method:**

Fifteen clinicians who work with children with ADHD and sleep difficulties in the United Kingdom participated in semistructured qualitative interviews. Data were analysed using a reflexive thematic analysis approach to generate and guide the content of digital sleep awareness training.

**Results:**

Four core themes were developed: ‘It's a Problem’ highlighted the extent to which children with ADHD were reported to struggle with sleep difficulties and the impact this has on the child and family. Clinicians also discussed the difficulty they had in finding evidence‐based information they could share with caregivers. With little to no formal training, most of the advice they gave came from ‘learning on the job’. When discussing ADHD specific sleep difficulties and disorders, clinicians reflected on their own ‘insight into limitations of knowledge’. ‘Learning for practice’ highlighted the divergence in the methods of learning preferred by clinicians, despite convergence of learning content needed.

**Conclusions:**

Sleep problems in children with ADHD are common, and clinicians often struggle to support these due to lack of formal training. There is a need for accessible, authoritative training for UK practitioners who work with children with ADHD.

## Summary

- When asked about their training needs on advising and supporting families of children with ADHD and sleep difficulties, clinicians confirm that sleep difficulties are a problem.
- Clinicians report that due to a lack of formal training, they often have to learn on the job and highlight limitations to their knowledge on sleep.
- Clinicians suggest that future training needs to be briefly delivered in bite‐sized sessions, probably in a digital format and from a trusted source.
- Lack of knowledge on sleep may lead to diagnostic over‐shadowing as well as confusion about ADHD risk versus risks associated with sleep problems.

## 1 Introduction

Attention deficit hyperactivity disorder (ADHD) is a neurodevelopmental disorder characterised by inattention and/or hyperactivity and impulsivity that is persistent and causes substantial impairment in functioning (Koutsoklenis and Honkasilta 2023). The global prevalence of ADHD varies, but most rigorous estimates report a prevalence of about 5% in children (Cortese et al. 2023). Symptoms of ADHD often begin in early childhood, at critical developmental time periods (Sonuga‐Barke and Halperin 2010), and are one of the most common reasons children are referred to mental health services (Hansen et al. 2021). Indeed, within the United Kingdom, the demand for care is significantly outstripping the capacity of services (French et al. 2023). Within the United Kingdom, ADHD service configuration and delivery vary widely, including community paediatrics, child and adolescent mental health services, CAMHS and occasionally local government.

ADHD is a highly co‐morbid disorder (Reale et al. 2017) with 7.5% of children presenting with a sleep disorder diagnosis and 47.5% being prescribed sleep medication (Ahlberg et al. 2023). However, there is no requirement for commissioning of therapeutic sleep services for children within the UK National Health Service, and so, in practice, provision is patchy. Indeed, high nocturnal activity and disordered sleep were defining characteristics of ‘hyperkinetic reaction in childhood’ or ‘attention deficit disorder’ in earlier versions of the DSM (Sadeh et al. 2006; Spruyt and Gozal 2011).

While no longer included as core symptoms of ADHD (Koutsoklenis and Honkasilta 2023), our focus on sleep problems in children with ADHD is important (French et al. 2023). First, sleep disorders can exacerbate core ADHD symptoms such as inattention, impulsivity and hyperactivity (Wajszilber et al. 2018). Second and importantly, treatment of sleep disorders can improve ADHD symptom expression (Becker 2020; Sciberras et al. 2020). Third, sleep complaints in children with ADHD exacerbate common co‐morbidities such as conduct disorder and anxiety disorder (Cortese et al. 2013). Finally, sleep complaints place considerable burden on parents or carers, often directly affecting their own sleep, mental health and quality of life (Martin et al. 2021; Peasgood et al. 2021). In a recent interview study, parents of children with ADHD reported struggles with initiating sleep, emotional distress impacting sleep, early waking despite late sleep onset and the behavioural and emotional consequences of poor sleep (Bondopadhyay et al. 2024).

Effective identification and management of sleep disorders in children with ADHD are therefore warranted and necessary, as part of a holistic and systemic management approach. Nonpharmacological interventions are recommended as a first‐line approach to address sleep difficulties and their underlying disorders in young people (Hornsey et al. 2025). However, understanding of and competence in identifying sleep disorders in children with ADHD are lacking (Reynolds et al. 2023; Sciberras et al. 2022). Sleep training for professionals has been neglected in professional curricula. King et al. (2021) concluded from their scoping review that the referral burden on health care systems for managing routine sleep disorders could be alleviated by educating a wider range of staff to diagnose and manage patients with sleep health issues. In line with the recommendations from King et al. (2021), the specific aim of this study was to explore, for the first time, the experiences of UK clinicians who work with children with ADHD and sleep difficulties, identify their unmet needs and, ultimately, inform a brief digital educational package for professionals about sleep difficulties in children with ADHD.

## 2 Methodology

### 2.1 Study Design

This qualitative interview study explores the experiences of the UK clinicians who work with children who have ADHD and sleep difficulties. The study formed part of the Digital Support for Children with ADHD (DISCA) research programme (https://www.discasleep.org.uk/) that aims to help parents, carers and health professionals manage sleep problems in children with ADHD.

### 2.2 Participants

Fifteen multidisciplinary clinicians (*n* = 12 female, *n* = 3 male) were recruited via study adverts distributed through clinical networks in the United Kingdom and advertised via the DISCA website. The DISCA study is a series of work packages that ultimately aims to develop and evaluate a digital sleep intervention to support parents of children with ADHD and a separate digital intervention to support clinicians. Eligibility criteria for this study included experience of working with families of children with ADHD and sleep difficulties. There were no exclusion criteria. As can be seen in Table 1, participants had on average more than 13 years of working with children with ADHD. They were predominantly nurses including a nurse consultant but also included other medical and nonmedical staff. They were predominantly female and worked mostly with children aged 6–18 years of age.

**TABLE 1:** Participant characteristics.

Participant	Clinical experience (years)	Job role	Gender	Client age range
Participant 1	4 years	Psychologist	Female	6–18 years
Participant 2	10+ years	Nurse	Male	6–18 years
Participant 3	10 years	CAMHS nurse	Female	5–18 years
Participant 4	15+ years	ADHD nurse	Female	6–18 years
Participant 5	30 years	Consultant nurse	Female	5–18 years
Participant 6	8 years	Paediatrician (trainee consultant)	Male	Children up to the age of 18
Participant 7	25 years	CAMHS nurse	Female	5–18 years
Participant 8	NA	CAMHS nurse	Female	6–18 years
Participant 9	25 years	Paediatric nurse	Female	6–18 years
Participant 10	15+ years	Advanced ADHD nurse	Male	Children and adults
Participant 11	10+ years	Sleep coach	Female	Children and adults
Participant 12	5+ years	Practice manager	Female	4–18 years
Participant 13	29 years	Consultant nurse	Female	6–18 years
Participant 14	3 years	Sleep practitioner	Female	6–18 years
Participant 15	9 years	CAMHS consultant	Female	6–18 years

Abbreviation: NA, information not available.

### 2.3 Interview Procedure

Interviews were conducted over Microsoft Teams and lasted between 19 and 66 min (mean duration 39 min). Interviews covered a range of topics including experience of sleep problems in practice, misconceptions in sleep and ADHD, education received about sleep, standard operation practice within the clinics such as referral mechanism and perceived training needs. Interviews were recorded, transcribed verbatim and anonymised. Fifteen participants provided sufficiently rich and meaningful data to address the study questions. Participants were thanked for their time with a £20 gift voucher.

### 2.4 Data Analysis

A reflexive thematic analysis (Braun and Clarke 2006, 2012, 2013) with an inductive approach was chosen to identify patterns in how participants made sense of their own experiences. Rigour and transparency were ensured by using the six phases outlined by Braun and Clarke, with an awareness of common pitfalls in qualitative analysis (Payne 2004) and quality in research and reporting (Braun and Clarke 2023).

DD and CH oversaw the data collection and analysis process with weekly meetings to ensure theoretical engagement, and analytic processes were followed to assure analytic power and validity. Biweekly meetings were held between LS, DD and CH to discuss the interviews and the analytic process. Familiarisation with the data (Phase 1) was undertaken independently by LS and CH noting points of interest. In Phase 2, a code was assigned to quotes of interest, and in Phase 3, codes were combined into themes via an iterative process; LS and CH placed emphasis on developing themes that could lead to actionable insights for the development of training for ADHD and sleep clinicians while remaining open to the complexity of individual experiences and challenges that participants faced in their day‐to‐day work. During Phases 4 and 5, themes were reviewed by LS, CH and DD to study the convergence and divergence of the code clusters and to define and name themes. This iterative process was strengthened by the diverse perspectives of the research team, which facilitated interpretation of the themes from both clinical and research standpoints, with a focus on practical outcomes within a pragmatist paradigm. Phase 6, reporting of findings, is outlined within this paper which focuses on four themes.

### 2.5 Reflexivity Statement

As a multidisciplinary team of academics and clinicians, a pragmatist theoretical paradigm was used (Bacon 2012; Dures et al. 2010). This focused on practical research outcomes while also allowing researchers from diverse backgrounds to work cohesively on the analysis (Patton 2002).

LS, who led on data collection and analysis, focused on practical outcomes of the research and experiential knowledge, reflecting on her personal experiences and how this shaped both data collection and analysis. LS has first‐hand experience of primary care and ADHD services after receiving a diagnosis in adulthood; while also having professional experience of working in neurodevelopmental research and children's trauma care advocacy, LS brings her own thoughts, feelings and biases due to her personal and professional experiences. LS conducted all interviews and was mindful of her role as the interviewer and the potential influence on participant responses; as such, personal experiences were not discussed with participants. LS was conscious of creating a nonjudgmental space where participants were central in discussing their experiences, thoughts and needs towards a digital training intervention for clinicians.

During data analysis, CH and DD also reflected on their personal and professional experiences that may have contributed to shaping the data analysis. CH has worked for over 25 years in developmental, social paediatrics and children's sleep medicine with a well‐developed awareness of sleep disorders and the mismatch between need and service provision. She also provides training in clinical sleep medicine and so recognises the knowledge gap of professionals. DD has both a professional interest in effective and enjoyable parenting of children with ADHD and ASD, as well as lived experience of neurodiversity.

While great care was taken to maintain integrity and reduce bias, it would be impossible to remove all biases due to the nature of reflexive thematic analysis. LS, CH and DD were attentive to these experiences and undertook analysis with a mindful lens to reduce the impact this could have.

## 3 Results

**FIGURE 1:**
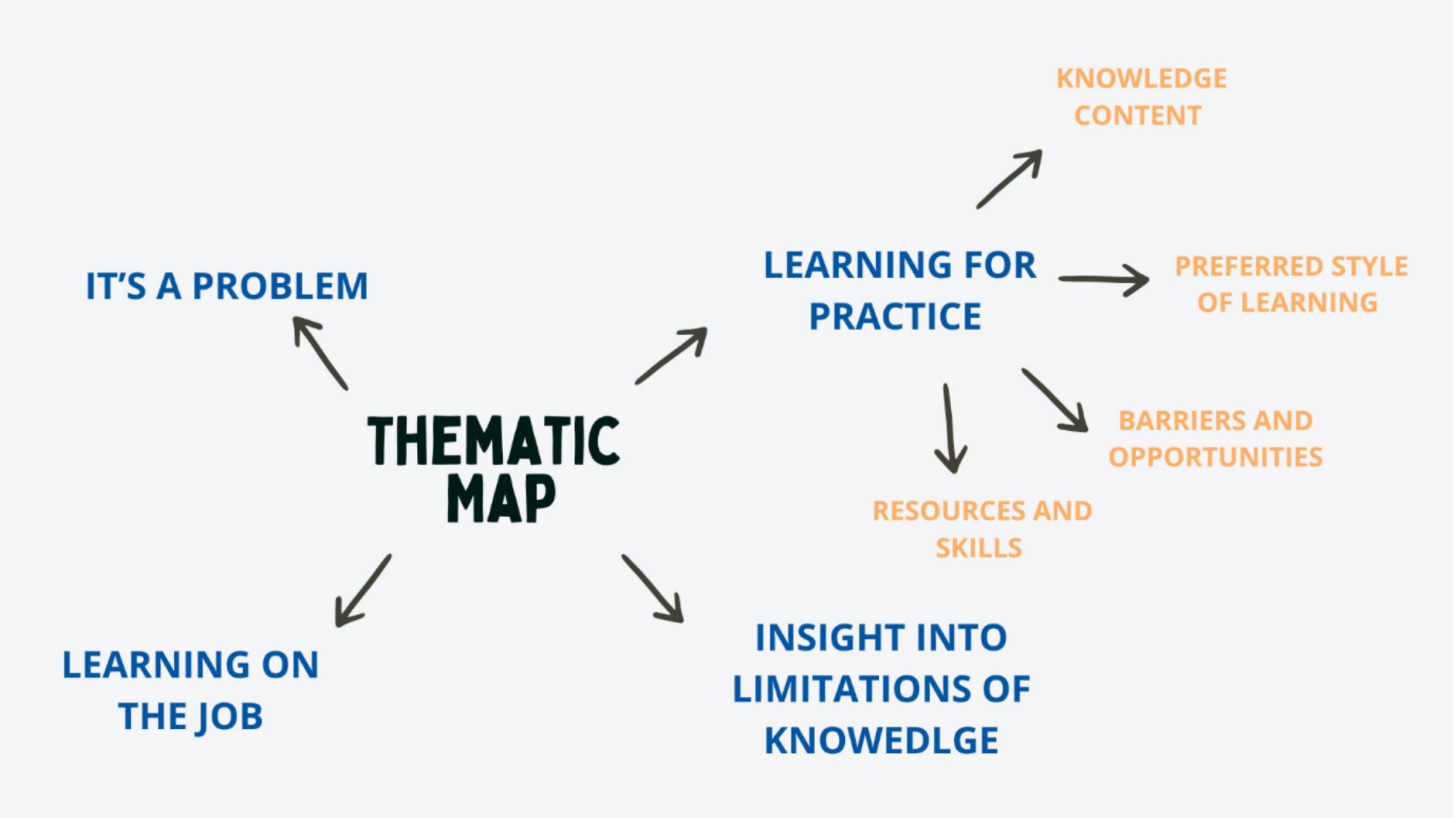
Themes and subthemes.

The four themes generated by this analysis are outlined in Figure 1. ‘It's a problem’ captures the extent to which sleep issues are present in practice and their impact on children with ADHD and their families. ‘Learning on the job’ reflects the lack of formal training clinicians have received on sleep in ADHD, despite it being a significant clinical problem. ‘Insight into limitations of knowledge’ reports the confidence clinicians have in their own ability to manage sleep problems in ADHD. ‘Learning for practice’ encompassed four subthemes ‘knowledge content, resources and skills’, ‘preferred style of learning’ and ‘barriers and opportunities’, which focus on the way that clinicians would like to receive training, the content they would find useful and the additional tools they need to continue to support families of children with ADHD.

### 3.1 It's a Problem

Participants reported that sleep is a common problem they support. This was particularly evident when the primary specialism was ADHD. Despite the wider scope of their work, they often reported sleep as the most significant issue that families sought help for.

ADHD and sleep are the bread and butter of what I do all day, every day. P9

I actually thought at one point I was a sleep nurse. P4

Participants noted the detrimental effect that a child's sleep can have on the rest of the family.

This is about the whole family the implications of a child's sleep and that the devastating consequences that this can have on families. P11

Sleep still is a very apparent issue and, the impact it has on isolating families and again, mental health of parents, and then their ability to care for their child, ermmm Employment. I think it is massive. P12

Participants also volunteered examples of risk behaviours related to children's sleep problems.

Some children, you know, somewhat higher risk as well that they would sort of leap out of their window because they're n‐no not able to sleep and you know, sit on the window ledge just looking at the sky where they find it peaceful. P15

They're going downstairs trying to get out the house or cooking things or, you know, just, doing things that are not sort of like safe, so then the parent does need to be awake to manage that. P14

The bidirectional relationship between sleep complaints and ADHD core symptoms was recognised and the need for clinicians to understand both sleep and ADHD to effectively manage difficult symptom expression.

But it's a two prong one isn't it because, yes, you know, we need to make sure that the ADHD is being managed effectively, erm, to help with the sleep, but then vice versa. ((laughs)). P14

### 3.2 Learning on the Job

A minority of participants had benefited from external training programmes in sleep disorder management. However, most participants reported a lack of formal training in sleep management and reported independent learning.

I've got a, erm, uh, a book that, that I got early on in my career that, erm, uh, which is called Back to Normal. Erm, and, erm, uh, and that's got some really useful, tips. P1

Learning on the job, by trial and error, or from supervision from more experienced colleagues was often reported.

I think I've kind really learned what I know on the job, on the job and practically giving advice and following these patients up, erm, and seeing how successful any interventions were. I have not been officially trained to support children with ADHD and sleep problems. P4

Actually, academically, not much. Erm, I think it's come more from working alongside people who have worked in neurodevelopmental stuff for years. So just sort of learning from them. And learning from the young people. P1

Participants also reported learning from families' experiences about what was successful in practice.

I've actually learned a lot from my families of my patients, as well. You know, tips and tricks and what the difficulties are. P4

### 3.3 Insight Into Limitations of Knowledge

Participants initially appeared to be confident in managing sleep problems and in knowing when and how to refer patients onto specialists. However, the question route included a prompt ‘To what extent do you feel confident identifying different types of sleep disorders’?, and this often caused a change in the narrative. At this point, most participants reported using general ‘lay’ knowledge about sleep as their framework for practice and recognised the limitation of ‘unknown unknowns’.

I think my knowledge is probably a bit, maybe a bit basic. So, I understand what a good bedtime routine and good bedtime sleep hygiene is. P7

Enhanced knowledge and understanding of sleep is a knowledge gap for CAMHS professionals, because it's not something we're ever actually taught. P13

Despite the prevalence of sleep problems experienced in everyday practice, most participants lacked understanding of sleep disorders.

I never knew there was actually um diagnostic categories for sleep, I had never really thought or understood the different range of the different diagnostic categories for sleep. P13

Clinicians were open to accepting opportunities to further develop their skills and confidence.

I know I would definitely, appreciate it. Um, because I think sleep is a really significant problem and we find it really difficult to manage. P6

### 3.4 Learning for Practice

This theme captures the two key factors that clinicians described when thinking about their training needs, namely, **knowledge content** and **preferred styles of learning**. Methods of delivery need to reflect the diversity of learning styles, while content needs to be grounded in evidence‐based theory. Clinicians also identified **resources and skills for practice** that they currently lack and **the barriers to opportunities** to engage with training successfully. All four subthemes are important in creating a well‐rounded picture of what clinicians recognise as needs in learning for practice.

### 3.5 Knowledge Content

Participants were able to identify specific training needs. Most evidently, there was a lack of knowledge about sleep disorders and their aetiology.

I would love to have a better understanding of sleep disorders generally. Um, in terms have different types of sleep disorders and diagnosis. P6

Clinicians were keen to learn but wanted to do so from an evidence‐based source that they could trust to give them confidence when working with children and their families.

Something that's consistent, that's something that's evidence base that's, you know, research and you know, people say, oh, okay, it's definitely true, then, because ((laughs)) there's been research, you know, paper on it. P14

I think, I mean, it's been a while since we've had an update. And I always like to have updates because the research moves on, ra‐ rapidly. P5

Participants also identified a better understanding of the role of hypnotic medication as a key learning requirement, as well as the secondary impact on sleep of ADHD medication.

Parents want medication too, you know, sleep medication to help their child sleep, and we're getting a lot more requests from GPs as well, to prescribe sort of melatonin. P3

Having a better understanding of how the ADHD medications could potentially be supportive in helping to manage sleep. Um, because it's really more focused on how we're managing the ADHD symptoms for the daytime. P6

However, it was not just more training on medications that clinicians felt would be beneficial; they also highlighted the difficulties they faced with managing parents' expectations around medication.

The go to is, we need Circadin we need Slenyto, can you help me? And that's really quite difficult when you can see these children are exhausted, you can see the parents are exhausted, sleep is an issue. But sort of saying, actually we need to do this first. P4

I cannot just give people melatonin and they'll get better. They need to also do some work at home as well. Um, and it's trying to encourage that work at home. P6

The struggles families face with technology use at bedtime were also discussed at length with a mixture of perspectives shared. Some participants had a balanced view of the benefits, and risks technology can pose to sleep in ADHD children, while others had a negative view of technology and focused more on the challenges it raises around bedtime.

Technology is, has really changed things. Blurred those boundaries. Both good and bad. You know, some of the strategies I'll use are technological based. P10

We do see a lot of young people that have big problems with gaming addiction, not wanting to come off the PlayStation. P13

Some of my teenagers are up doing their homework till 11 o'clock at night on a laptop. So then we're saying that on one hand, and then with the other hand, we're saying, don't use technology too late at night. It's almost like our patients can't win. P4

Participants recognised the need for evidence‐based information around technology and sleep to deliver support to families with confidence.

Just to sort of know the behavioural sleep side of stuff that what is actually around the screen usage, because different professionals say different things around sort of technology and usage. P12

### 3.6 Preferred Styes of Learning

There was a range of preferred learning styles identified by participants, but most focused on training that was interactive and able to hold their attention.

More of a visual‐ Something interactive, yeah, visual and interactive. P14

Short clips are always good (laughs). To keep my concentration. P8

Yeah, that more interactive and meaningful sort of [mm] approach rather than…speaking at it, you know, case studies are normally quite good, because you see different people's opinions. P1

It was important to some that training provided an opportunity for them to also engage in their own personal learning styles, such as being able to take notes, listen to an audio of the information.

I think um for me, personally, I prefer to watch somebody talk about it. Because I think tone of voice and everything just helps me to process that information and retain that information. Uh maybe backed up with written as well. And I definitely think online. P11

Podcasts are great actually, I love podcasts. P5

### 3.7 Barriers to Opportunities

While clinicians cast a spotlight on the unmet demand for ADHD‐specific sleep training, they also were clear about perceived barriers to accessing learning. Time was one of the biggest challenges with a common desire for ‘bite sized’ training that could fit in flexibly around busy schedules.

With how pressured, time is in, in healthcare at the moment, the only way that you'll get somebody to engage in it, is if it's in a really short bite size. P2

Clinicians also felt that training needed to be specific to their level of expertise and delivered from a credible source.

It's got to be new, it's got to be in depth, it's got to be niche. P10

Maybe it would in terms of believing that the person who was offering the training was educated enough to offer the training. P11

### 3.8 Resources for Practice

Clinicians shared the importance of having evidence‐based practical resources that could be used to support their practice.

Having somewhere realistic to signpost parents to, so when you were saying earlier about, you know developing digital content for families, having something that we can signpost families to, I think would be enormously helpful. P13

Clinicians also highlighted the importance of skills to manage difficult conversations around behaviour change with parents and to ‘empower’ parents to support their children.

I think it'd be useful to be able to have sort of that bit of a dialogue about the rationale as to why we're telling them to change. P2

Them knowing what the processes are for how to get help with sleep. And kind of empowering them to then help their children. P8

## 4 Discussion

This study explored, for the first time, the training needs of the UK practitioners working with children with ADHD and sleep difficulties. Four themes, one with four subthemes, were identified: (1) It's a problem, (2) learning on the job, (3) insights into limitations of knowledge and (4) learning for practice (which also included [4.1] knowledge content, [4.2] preferred style of learning, [4.3] barriers and opportunities and [4.4] resources and skills). Our findings provide important insights into the content that is needed within ADHD specific sleep training, the type of training that is needed to engage clinicians and the barriers they face to learning.

While this paper focused on exploring the training needs of the UK clinicians, it is important to reflect on the context of their practice. Clinicians consistently emphasised the intensity and impact of sleep‐related issues experienced by children with ADHD, which aligns with prior research findings (Bondopadhyay et al. 2024; French et al. 2023). This extended to accounts of risky behaviours at night that clinicians described mostly as sensation seeking (Pollak et al. 2019; Shoham et al. 2021). While risk‐taking is not unique to sleep issues in ADHD, there is a risk that diagnostic overshadowing may cause underlying sleep disorders to be ignored by clinicians failing to separate broader ADHD symptoms. It may also point to a gap in clinicians' training, where they struggle to distinguish general ADHD‐related risk behaviours from those specifically linked to sleep challenges. Clinicians also identified harms extending beyond the wellbeing of the index child to other household members. Similarities can be seen with French et al.'s (2023) theme ‘The cumulative effect of lack of sleep: impact on functioning and the wider family’ where parents reported their struggles with employment, mood and mental health. In summary, the theme ‘It's a problem’ highlighted clinicians' endorsement of sleep difficulties as a significant challenge in their everyday practice.

While qualitative studies have investigated the experiences of parents, leading to suggestions for further education for parents of ADHD children around their sleep difficulties (Bondopadhyay et al. 2024; French et al. 2023), we believe this is the first study to explore the training needs of the specialists who work with families of these children in the United Kingdom. The findings indicate that most clinicians acquire their sleep knowledge through hands‐on, practical learning and exposure in their roles. Clinicians sought answers to the sleep‐related questions posed by families in need of support and mostly worked from experience. The absence of formal, evidence‐based training on sleep issues in ADHD stands in sharp contrast to broader ADHD training, which typically follows established procedures within professional qualifications, such as in nursing. While there was recognition by clinicians of the value of experience, many expressed concerns about the reliability of their personal knowledge. Evidence‐based training from a trusted source was seen as a critical step in enhancing clinicians' ability to support families effectively and in strengthening their confidence.

Clinicians also reported a lack of confidence in their own knowledge about sleep disorders, despite being seen as specialists. Interestingly, this lack of knowledge was something that the health care professionals were keen to share (Meyer et al. 2021). It is possible that responses were influenced by the expressed aims of the study. Reflexive analysis of the data identified a pattern of responses across a few participants who initially expressed confidence in their knowledge until the interviewer posed a question on sleep disorders. At this point, clinicians who had initially felt confident in their sleep knowledge, recognised their knowledge limitations. The use of the term ‘disorder’ applied to sleep reframes ‘sleep difficulties or problems’ in a clinical diagnostic context. As noted by one such participant ‘I never knew there was actually um diagnostic categories for sleep, I suppose if I stop and think about it, then it‐ it‐ it's obvious’. This underlines the risks of ‘unknown unknowns’ when seeking to understand training needs.

‘Learning for practice’ provided valuable information to inform the content and delivery of future clinician training. In terms of training, clinicians felt they would benefit from better understanding of sleep disorders and how these relate to ADHD, the interplay between ADHD medications and sleep and indications for hypnotic use. A recurrent learning need was also a better understanding of how to manage and advise on technology use in the home both as a tool or barrier in sleep support. Importantly, clinicians emphasised that any content should be grounded in evidence‐based theory from experts in the field.

Clinicians identified different personal learning styles and often acknowledged the diversity of learning approaches among peers. There was a consensus preference for interactive learning content. While not formally explored, several clinicians identified their own neurodiversity as an obstacle to learning and suggested ‘bite sized’ learning approaches as a possible solution.

Barriers to training were lack of time, particularly in busy NHS clinics where managing waiting lists is a priority. For this reason, short online training was seen as an accessible option. Clinicians also wanted training suited to their skill level and specific to their patient population. Credibility of the training resource was also seen as important, alongside evidence‐based content. Clinicians expressed a wish for evidenced‐based information resources for families to support practice. They also wanted skills‐based training to empower parents to manage behaviour change in their child and to better manage difficult conversations such as mismatched expectations for sleep and ADHD medications and resistance from parents to try a different approach.

Strengths of this study included a flexible approach to provide accessible interviews at times to suit the clinicians. This was critical in engaging ADHD and sleep specialists who work for busy services. Having time for the interviews allowed rapport to be established and participants to discuss their perspectives on the intersection of ADHD and sleep. This participant population was also well situated to provide contextual understanding of training needs whilst factoring in real‐world barriers to learning. Furthermore, a rigorous methodological approach was used by an experienced multi‐disciplinary team.

Limitations included lack of data on whether clinicians had an ADHD diagnosis themselves, or if they had children with ADHD. However, several spoke about their personal experiences with ADHD. It is possible that personal experiences may influence professional outlook on training requirements, and in the future, this information may be worth collecting and considering in relation to the data. Finally, it was not possible to explore in detail how clinicians respond to the sleep difficulties parents face, which is the focus of a separate paper currently in development.

This study provides important implications for future clinical training and research. Firstly, training in sleep specific ADHD difficulties is something that clinicians feel is urgently needed and actively wanted. Secondly, any training needs to be evidence‐based and from a trusted source. Thirdly, training must be available in short, engaging, interactive segments. Fourth, content for training should focus on sleep disorders and difficulties specific to ADHD, sleep and ADHD medications and the impact of technology at bedtime. Fifth, training would be enhanced by matched resources for parents as well as skills‐based training to support clinicians to navigate difficult conversations with families. This research suggests that the development of sleep specific ADHD training for professionals is warranted, and future research would benefit from evaluating the effectiveness of the training that is developed.

## Author Contributions

**Lucy Smith:** investigation, writing – original draft, methodology, validation, writing – review and editing, visualization, project administration, formal analysis. **David Daley:** conceptualization, investigation, writing – original draft, methodology, writing – review and editing, data curation. **Samuele Cortese:** conceptualization, investigation, writing – original draft, methodology, writing – review and editing. **Catherine M. Hill:** conceptualization, investigation, writing – original draft, methodology, writing – review and editing, formal analysis.

## Funding

This study/project is funded by the NIHR PGfAR NIHR203684.

## Ethics Statement

Full ethical approval for this study was granted by S3 Research Ethics Committee, through Nottingham Trent University, application ID 2190028 on 14.02.2023. All clinicians who participated in this study provided full written consent as well as verbal reconsent on the day of their interview.

## Consent

The authors have nothing to report.

## Conflicts of Interest

D.D. has in past provided educational talks for Medice and Takeda. He has also reported grants, personal fees and nonfinancial support from Takeda, Medice, ACAMH Learn and the New Forest Parenting Programme, book royalties from the sale of a self‐help version of the New Forest Parenting Programme and compensation for the provision of training and supervision in the New Forest Parenting Programme. He has recevied travel support from the World Federation of ADHD, and is a scientific advisor for Assembly and Neuronotion. S.C. has declared reimbursement for travel and accommodation expenses from the Association for Child and Adolescent Central Health (ACAMH) in relation to lectures delivered for ACAMH, the Canadian AADHD Alliance Resource and the British Association of Psychopharmacology, Healthcare Convention. All other authors report no conflicts of interest.

## Data Availability

The data that support the findings of this study are available on request from the corresponding author. The data are not publicly available due to privacy or ethical restrictions.

## References

[cch70254-bib-0001] Ahlberg, R. , M. Garcia‐Argibay , M. Taylor , et al. 2023. “Prevalence of Sleep Disorder Diagnoses and Sleep Medication Prescriptions in Individuals With ADHD Across the Lifespan: A Swedish Nationwide Register‐Based Study.” BMJ Mental Health 26, no. 1: e300809. 10.1136/bmjment-2023-300809. PMC10577710 37657817

[cch70254-bib-0002] Bacon, M. 2012. Pragmatism: An Introduction, Polity.

[cch70254-bib-0003] Becker, S. P. 2020. “ADHD and Sleep: Recent Advances and Future Directions.” Current Opinion in Psychology 34: 50–56. 10.1016/j.copsyc.2019.09.006. 31629217 PMC7082190

[cch70254-bib-0004] Bondopadhyay, U. , J. McGrath , and A. N. Coogan . 2024. ““Tell Me More About Your Child's Sleep”: A Qualitative Investigation of Sleep Problems in Children With ADHD.” Behavioral Sleep Medicine 22, no. 3: 298–307. 10.1080/15402002.2023.2253947. 37665076

[cch70254-bib-0005] Braun, V. , and V. Clarke . 2006. “Using Thematic Analysis in Psychology.” Qualitative Research in Psychology 3, no. 2: 77–101. 10.1191/1478088706qp063oa.

[cch70254-bib-0006] Braun, V. , and V. Clarke . 2012. “Thematic Analysis.” In APA Handbook of Research Methods in Psychology, Vol 2: Research Designs: Quantitative, Qualitative, Neuropsychological, and Biological, 57–71, American Psychological Association. 10.1037/13620-004.

[cch70254-bib-0007] Braun, V. , and V. Clarke . 2013. Successful Qualitative Research: A Practical Guide for Beginners, 1–400, SAGE Publications Ltd.

[cch70254-bib-0008] Braun, V. , and V. Clarke . 2023. “Is Thematic Analysis Used Well in Health Psychology? A Critical Review of Published Research, With Recommendations for Quality Practice and Reporting.” Health Psychology Review 17, no. 4: 695–718. 10.1080/17437199.2022.2161594. 36656762

[cch70254-bib-0009] Cortese, S. , T. E. Brown , P. Corkum , et al. 2013. “Assessment and Management of Sleep Problems in Youths With Attention‐Deficit/Hyperactivity Disorder.” Journal of the American Academy of Child and Adolescent Psychiatry 52, no. 8: 784–796. 10.1016/j.jaac.2013.06.001. 23880489

[cch70254-bib-0032] Cortese, S. , M. Song , L. C. Farhat , et al. 2023. “Incidence, Prevalence, and Global Burden of ADHD from 1990 to 2019 Across 204 Countries: Data, with Critical Re‐Analysis, from the Global Burden of Disease Study.” Molecular Psychiatry 28, no. 11: 4823–4830. 37684322 10.1038/s41380-023-02228-3

[cch70254-bib-0010] Dures, E. , N. Rumsey , M. Morris , and K. Gleeson . 2010. “Mixed Methods in Health Psychology.” Journal of Health Psychology 16: 332–341. 10.1177/1359105310377537. 20978152

[cch70254-bib-0011] French, B. , E. Quain , J. Kilgariff , J. Lockwood , and D. Daley . 2023. “The Impact of Sleep Difficulties in Children With Attention Deficit Hyperactivity Disorder on the Family: A Thematic Analysis.” Journal of Clinical Sleep Medicine 19, no. 10: 1735–1741. 10.5664/jcsm.10662. 37786381 PMC10545997

[cch70254-bib-0012] Hansen, A. S. , C. H. Christoffersen , G. K. Telléus , and M. B. Lauritsen . 2021. “Referral Patterns to Outpatient Child and Adolescent Mental Health Services and Factors Associated With Referrals Being Rejected. A Cross‐Sectional Observational Study.” BMC Health Services Research 21, no. 1: 1063. 10.1186/s12913-021-07114-8. 34625073 PMC8501731

[cch70254-bib-0013] Hornsey, S. J. , C. J. Gosling , L. Jurek , et al. 2025. “Umbrella Review and Meta‐Analysis: The Efficacy of Nonpharmacological Interventions for Sleep Disturbances in Children and Adolescents.” Journal of the American Academy of Child and Adolescent Psychiatry 64, no. 3: 329–345. 10.1016/j.jaac.2024.10.015. 39608635

[cch70254-bib-0014] King, S. , R. Damarell , L. Schuwirth , A. Vakulin , C. L. Chai‐Coetzer , and R. D. McEvoy . 2021. “Knowledge to Action: A Scoping Review of Approaches to Educate Primary Care Providers in the Identification and Management of Routine Sleep Disorders.” Journal of Clinical Sleep Medicine 17, no. 11: 2307–2324. 10.5664/jcsm.9374. 33983109 PMC8636382

[cch70254-bib-0015] Koutsoklenis, A. , and J. Honkasilta . 2023. “ADHD in the DSM‐5‐TR: What Has Changed and What Has Not.” Frontiers in Psychiatry 13: 1064141. 36704731 10.3389/fpsyt.2022.1064141 PMC9871920

[cch70254-bib-0016] Martin, C. A. , N. Papadopoulos , N. Rinehart , and E. Sciberras . 2021. “Associations Between Child Sleep Problems and Maternal Mental Health in Children With ADHD.” Behavioral Sleep Medicine 19, no. 1: 12–25. 10.1080/15402002.2019.1696346. 31760782

[cch70254-bib-0017] Meyer, A. N. , T. D. Giardina , L. Khawaja , and H. Singh . 2021. “Patient and Clinician Experiences of Uncertainty in the Diagnostic Process: Current Understanding and Future Directions.” Patient Education and Counseling 104, no. 11: 2606–2615. 34312032 10.1016/j.pec.2021.07.028

[cch70254-bib-0018] Patton, M. Q. 2002. “Two Decades of Developments in Qualitative Inquiry: A Personal, Experiential Perspective.” Qualitative Social Work Research and Practice 1, no. 3: 261–283. 10.1177/1473325002001003636.

[cch70254-bib-0019] Payne, S. 2004. “Designing and Conducting Qualitative Studies.” In Health Psychology in Practice, 126–149, John Wiley & Sons, Ltd. 10.1002/9780470694008.ch7.

[cch70254-bib-0020] Peasgood, T. , A. Bhardwaj , J. E. Brazier , et al. 2021. “What Is the Health and Well‐Being Burden for Parents Living With a Child With ADHD in the United Kingdom?” Journal of Attention Disorders 25, no. 14: 1962–1976. 10.1177/1087054720925899. 32552265 PMC8527548

[cch70254-bib-0021] Pollak, Y. , T. J. Dekkers , R. Shoham , and H. M. Huizenga . 2019. “Risk‐Taking Behavior in Attention Deficit/Hyperactivity Disorder (ADHD): A Review of Potential Underlying Mechanisms and of Interventions.” Current Psychiatry Reports 21, no. 5: 33. 10.1007/s11920-019-1019-y. 30903380

[cch70254-bib-0022] Reale, L. , B. Bartoli , M. Cartabia , et al. 2017. “Comorbidity Prevalence and Treatment Outcome in Children and Adolescents With ADHD.” European Child & Adolescent Psychiatry 26, no. 12: 1443–1457. 10.1007/s00787-017-1005-z. 28527021

[cch70254-bib-0023] Reynolds, A. , A. Spaeth , L. Hale , et al. 2023. “Pediatric Sleep: Current Knowledge, Gaps, and Opportunities for the Future.” Sleep 46, no. 7: 1–21. 10.1093/sleep/zsad060. PMC10334737 36881684

[cch70254-bib-0024] Sadeh, A. , L. Pergamin , and Y. Bar‐Haim . 2006. “Sleep in Children With Attention‐Deficit Hyperactivity Disorder: A Meta‐Analysis of Polysomnographic Studies.” Sleep Medicine Reviews 10, no. 6: 381–398. 10.1016/j.smrv.2006.03.004. 16846743

[cch70254-bib-0026] Sciberras, E. , M. Mulraney , N. Hayes , et al. 2022. “A Brief Clinician Training Program to Manage Sleep Problems in ADHD: What Works and What Do Clinicians and Parents Think?” Sleep Medicine 89: 185–192. 10.1016/j.sleep.2021.04.007. 34001454

[cch70254-bib-0027] Sciberras, E. , M. Mulraney , F. Mensah , F. Oberklaid , D. Efron , and H. Hiscock . 2020. “Sustained Impact of a Sleep Intervention and Moderators of Treatment Outcome for Children With ADHD: A Randomised Controlled Trial.” Psychological Medicine 50, no. 2: 210–219. 10.1017/S0033291718004063. 30654852

[cch70254-bib-0028] Shoham, R. , E. Sonuga‐Barke , I. Yaniv , and Y. Pollak . 2021. “What Drives Risky Behavior in ADHD: Insensitivity to Its Risk or Fascination With Its Potential Benefits?” Journal of Attention Disorders 25, no. 14: 1988–2002. 10.1177/1087054720950820. 32854554

[cch70254-bib-0029] Sonuga‐Barke, E. J. S. , and J. M. Halperin . 2010. “Developmental Phenotypes and Causal Pathways in Attention Deficit/Hyperactivity Disorder: Potential Targets for Early Intervention?” Journal of Child Psychology and Psychiatry 51, no. 4: 368–389. 10.1111/j.1469-7610.2009.02195.x. 20015192

[cch70254-bib-0030] Spruyt, K. , and D. Gozal . 2011. “Pediatric Sleep Questionnaires as Diagnostic or Epidemiological Tools: A Review of Currently Available Instruments.” Sleep Medicine Reviews 15, no. 1: 19–32. 10.1016/j.smrv.2010.07.005. 20934896 PMC3088759

[cch70254-bib-0031] Wajszilber, D. , J. A. Santiseban , and R. Gruber . 2018. “Sleep Disorders in Patients With ADHD: Impact and Management Challenges.” Nature and Science of Sleep 10: 453–480. 10.2147/NSS.S163074. PMC6299464 30588139

